# Measuring Research Impact in a Health Service Is a Worthy but Complex Goal; A Response to Recent Commentaries

**DOI:** 10.34172/ijhpm.2023.8334

**Published:** 2023-11-28

**Authors:** Amy Brown, Tilley Pain, Alexandra Edelman, Sarah Larkins, Gillian Harvey

**Affiliations:** ^1^Townsville Hospital and Health Service, Townsville, QLD, Australia; ^2^James Cook University, Townsville, QLD, Australia; ^3^Menzies School of Health Research, Charles Darwin University, Alice Springs, NT, Australia; ^4^Flinders University, Adelaide, SA, Australia

 We thank the authors responding to our recent article describing our research impact evaluation at a regional Australian hospital and health service.^1^ The five considered commentaries contribute to the continued and much needed dialogue about embedded research integrated into health systems. We deliberately use “embedded” and “integrated” to infer research is fixed firmly (embedded) and incorporated (integrated) into health systems so that research in health systems achieves scholarly rigour and importantly practice change. In response, we address each commentary individually and provide context for our health service moving forward.

 Ramanathan argues the crux of an impact evaluation is to report the benefits and impacts of the research undertaken, and rightly note this evidence was difficult to identify in our study.^2^ The impact examples in our study were identified through interviews and document review, leading us to recommend Townsville Hospital and Health Service (THHS) and similar health services, prospectively and systematically collect impact data including patient outcomes alongside traditional research outputs. Systematic collection will facilitate increasingly robust research impact evaluations. Ramanathan states our study measured research activity and productivity, not impact; and suggests field weighted citation indexes could have been reported. We agree, while also emphasising that healthcare delivery organisations must also identify and collect relevant clinical and workforce-related indicators of research impact.

 We thank Abramo and D’Angelo’s for bibliometric analysis of THHS data and are pleased to note the increasing trends from THHS.^3^ The scholarly impact measured by bibliometric analysis is certainly an important aspect, but again we emphasise the importance of looking beyond measures of research activity to indicators of impact that incentivise knowledge translation pathways into clinical practice. Future efforts to define impact for our healthcare organisation will align with the social impact they describe.

 Williams and Fernandes surmised three lessons from our study: (1) achieving a shared definition and expectation of research, (2) the importance of stakeholder engagement, and (3) enabling research across a system.^4^ They correctly noted the diverse expectations of research among key informants at THHS which can (and should) be ameliorated by stakeholder engagement with management to clarify the purpose of investing in research. We wholeheartedly agree on the importance of stakeholder engagement, particularly managers and community, to support the translation of evidence into practice. As Williams and Fernandes state, without a shared definition and expectation of research, research impacts will be equally hard to define and measure.^4^ We also agree with the suggested approach for individuals from all clinical and management disciplines to develop research skills such as critical appraisal, community engagement and analysis of financial cost and health utilisation; noting we have made recent progress at our health service.

 Hanney commented on the specific and broader impacts identified in their evidence synthesis on research engagement and healthcare performance. The specific impacts refer to rapid implementation of research which we consider the most relevant for research conducted in healthcare organisations.^5^ Hanney argues the research successes and challenges during the COVID-19 pandemic demonstrate the importance of embedded research within health systems. At THHS, COVID-19 had an overall negative impact on research, with an institutional pause on research (except COVID-19-related studies or clinical trials of limited treatment options). Researchers and research administration staff were re-deployed to the COVID-19 front line creating a backlog, particularly in ethical and governance approvals. This shift in resources underscores the importance of integrating workforce and infrastructure investments into efforts to embed research in a health service. We also call for improved integration of research and healthcare, with appropriate resourcing, to provide better patient outcomes; but highlight the regulative barriers often faced by health service administrators who are accountable to healthcare performance indicators and financing models that do little to incentivise research and translation pathways.

 Archibald poses the question “why does embedded research matter” and argues for its integration into standard care.^6^ Their suggested pluralistic view of research and evidence includes systematic approaches to research and translation, building research capacity and literacy, and research investment and priority alignment. The call for a research-ready, learning health system presents an opportunity for health systems to actively and rapidly improve care delivery. THHS has recently invested in a data lab to enable rapid and streamlined access to data, particularly from the electronic medical records, for approved research and quality improvement activities. While in its infancy, we consider this a step towards improved systematic data collection and access.

 Measuring impact of research embedded in a health service remains a worthy but complex goal. Future impact evaluations in health care organisations can draw from the important lessons collated in the commentaries, including integrating systematic data collection processes for impact assessment, engaging with key stakeholders to clarify the research priorities, using bibliometrics to measure scholarly activity, and re-framing health care organisations as learning health systems. We draw attention to the need to consider the broader regulative contexts in which healthcare organisations operate that shape incentives not only for investing in research and knowledge translation but also the conduct of research impact evaluations. It is only through consciously embracing these multi-faceted process and context elements framing research investment and evaluation that healthcare organisations can improve patient care through locally responsive, quality research.

## Ethical issues

 Not applicable.

## Competing interests

 AB and TP are current employees of the THHS. AE was an employee of the THHS at the time of the original study.
